# *Bartonella* Species in Raccoons and Feral Cats, Georgia, USA

**DOI:** 10.3201/eid1907.130010

**Published:** 2013-07

**Authors:** Jusun Hwang, Nicole L. Gottdenker

**Affiliations:** Author affiliation: University of Georgia, Athens, Georgia, USA

**Keywords:** Bartonella, bacteria, raccoons, feral cats, zoonoses, urban ecology, urbanized barrier island, Georgia, United States

**To the Editor:** *Bartonella* spp. are vector-borne, facultative, intracellular bacteria that infect mammalian erythrocytes and endothelial cells and might cause chronic bacteremia and asymptomatic infections in reservoir hosts (*1*). There are currently 30–40 identified *Bartonella* species (*2*), and 14 of them are zoonotic; they have a wide variety of reservoirs, including rodents, carnivores, and ungulates (*3*). This study describes 2 *Bartonella* species in an urban population of raccoons and compares these findings to *Bartonella* infection in sympatric feral cats (*Felis catus*).

Raccoons (*Procyon lotor*) (n = 37) were live-trapped (Tomahawk Life Trap Company, Tomahawk, WI, USA) in spring and summer of 2012 on St. Simons Island, an urbanized coastal barrier island in Georgia in the southeastern United States (31°9′40″N, 81°23′13″W). The island is characterized by beach, salt marsh, forest, freshwater slough, and extensive residential developments. Raccoons were anesthetized with 20 mg/kg ketamine (Aveco Co., Fort Dodge, IA, USA) and 4 mg/kg xylazine (Mobay Corp., Shawnee, KS, USA), and blood was collected from the jugular vein into tubes containing EDTA. Feral cat blood samples (n = 37) from trap-neuter programs were collected by local veterinarians on St. Simons Island. Institutional Animal Care and Use Committee (A2011 03-042-Y2-A2) and Georgia Department of Natural Resources wildlife permits (29-WBH-12-100) were obtained before sampling.

DNA was extracted from blood by using a commercial DNA extraction kit (Quick-gDNA MiniPrep; Zymo Research Corp., Orange, CA, USA). Extracted DNA was used to amplify the 16S–23S rRNA intergenic spacer region of *Bartonella* spp. by nested PCR. For outer PCR, we used primers QHVE-1 (5′-TTCAGATGATGATCCCAAGC-3′) and QHVE-3 (5′-AACATGTCTGAATATATCTTC-3′) (*4*,*5*). PCR was performed with an initial incubation for 2 min at 94°C; 35 cycles of denaturation at 94°C for 30 s, primer annealing at 52°C for 30 s, and elongation at 72°C for 60 s; and a final incubation at 72°C for 6 min.

Nested PCR was performed by using primers QHVE-12 (5′-CCG GAG GGC TTG TAG CTC AG-3′) and QHVE-14b (5′-CCT CACAAT TTC AAT AGA AC-3′) (*4*). Nested PCR conditions were identical to those for the outer PCR, except for the annealing temperature, which was 55°C. Positive amplicons were separated by electrophoresis on a 1.2% agarose gel and purified by using the QIAquick PCR Purification Kit (QIAGEN, Valencia, CA, USA).

Purified DNA amplicons (400–600 bp) were sequenced by using an ABI automated sequencer (Applied Biosystems, Foster City, CA, USA). Intergenic spacer sequences from raccoon isolates were aligned with reported *Bartonella* species sequences in GenBank by using the ClustalW algorithm (*6*). A phylogenetic tree of the sequences was constructed by using neighbor-joining methods and maximum composite likelihood distances. Data were resampled 1,000 times to generate bootstrap values by using MEGA5 (*7*).

Of 74 samples analyzed (37 raccoon, 37 feral cat), 16 (43%) raccoon samples and 18 (48%) feral cat samples were positive for *Bartonella* spp. by PCR. Thirteen positive raccoon samples and 16 positive feral cat samples were sequenced. Twelve positive raccoon samples and 13 positive feral cat samples contained *Bartonella henselae. B. koehlerae* was amplified from 1 feral cat sample and 1 raccoon sample (99% sequence homology with a *B. koehlerae* sequence, GenBank accession no. AF312490). Two feral cat samples were identified as containing *B. clarridgeiae* and showed 98% and 100% sequence homology with a *B. clarridgeiae* sequence (GenBank accession no. AF167989) (Table; Figure, Appendix).

**Table:** *Bartonella spp.–*positive raccoons and feral cats identified by PCR and sequencing of DNA extracted from whole blood, Georgia, USA*

*Bartonella* species	Raccoon (*Procyon lotor*)	Feral cat (*Felis catus*)
*B. henselae*	12/37	13/37
*B. koehlerae*	1/37	1/37
*B. clarridgeiae*	0/37	2/37

*Values are no. positive/no. tested.

**Figure:**
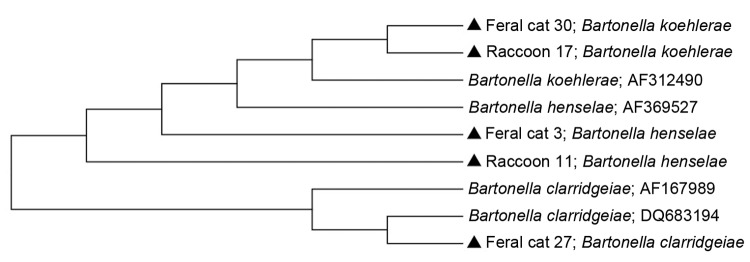
Phylogenetic tree of intergenic spacer region genes of *Bartonella* species inferred by the neighbor-joining method using the maximum composite likelihood method. Samples from this study are indicated by a solid triangle. GenBank accession numbers are indicated after species name.

This study identified *B. henselae and B. koehlerae* in feral cat and raccoons and *B. clarridgeiae* in feral cats. Our results are useful because raccoons are potential reservoir hosts of zoonotic *B. henselae* and *B. koehlerae*, in addition to *B. rochalimae*, and there could be cross-species transmission of *Bartonella* spp. between feral cats and raccoons.

Among reservoir hosts for *Bartonella* species, rodents and cats have been the most extensively studied. Rodents harbor 11 *Bartonella* species (*3*). Cats are the principal reservoirs of *B. clarridgeiae,* which causes endocarditis in humans, and *B. henselae*, which causes cat-scratch disease. However, little is known about *Bartonella* spp. infections in raccoons; there is only 1 report of *B. rochalimae* in raccoons in California (*8*).

In this study, a relatively high proportion of raccoons were infected with *B. henselae*, implying that there is spillover of *B. henselae* from feral cats to raccoons or that raccoons are another active reservoir for *B. henselae*. *B. clarridgeiae* and *B. koehlerae* are also zoonotic; cats are primary reservoirs, and humans and dogs are accidental hosts (*1*). However, *B. clarridgeiae* was recently detected in rodent fleas in China (*9*) and *B. koehlerae* was isolated from feral pigs from the southeastern United States (*10*), suggesting that these pathogens also have multiple reservoir species.

Clarifying whether *Bartonella* infections in raccoons are caused by spillover from feral cats needs further study. Additional samples from raccoons and other species in urbanized and undeveloped habitats with different host species composition (e.g., cat-free environment) might enable further *Bartonella* spp. characterization in wildlife. We suspect urban raccoons and feral cats play a major role in *Bartonella* spp. transmission.
